# HIF-1α-HPRT1 axis promotes tumorigenesis and gefitinib resistance by enhancing purine metabolism in EGFR-mutant lung adenocarcinoma

**DOI:** 10.1186/s13046-024-03184-8

**Published:** 2024-09-30

**Authors:** Pengyu Geng, Fei Ye, Peng Dou, Chunxiu Hu, Jiarui He, Jinhui Zhao, Qi Li, Miao Bao, Xiangnan Li, Xinyu Liu, Guowang Xu

**Affiliations:** 1 State Key Laboratory of Medical Proteomics, CAS Key Laboratory of Separation Science for Analytical Chemistry, Dalian Institute of Chemical Physics, Chinese Academy of Sciences, Dalian, Liaoning Province 116023 China; 2 Liaoning Province Key Laboratory of Metabolomics, Dalian, Liaoning Province 116023 China; 3 The First Affiliated Hospital of Zhengzhou University, Zhengzhou, Henan Province 450052 China; 4 Clinical Laboratory, The Second Hospital of Dalian Medical University, Dalian, Liaoning Province 116023 China; 5 Department of Medical Oncology, The First Affiliated Hospital of Xi’an Medical University, Xi’an, Shanxi Province 710082 China

**Keywords:** LUAD, EGFR, Purine metabolism, HIF-1α, HPRT1

## Abstract

**Background:**

The mutations of oncogenic epidermal growth factor receptor (EGFR) is an important cause of lung adenocarcinoma (LUAD) malignance. It has been knowm that metabolic reprogramming is an important hallmark of malignant tumors, and purine metabolism is a key metabolic pathway for tumor progression and drug resistance, but its relationship with the EGFR-mutant LUAD is unclear.

**Methods:**

Metabolic reprogramming was studied through capillary electrophoresis-time of flight mass spectrometry (CE-TOF/MS)-based metabolic profiling analysis. Cell proliferation in vitro was evaluated by EdU staining and cell cycle assay. Tumorigenicity in vivo was tested by subcutaneous tumor formation experiment in nude mice. The binding of hypoxia-inducible factor-1 alpha (HIF-1α) and hypoxanthine phosphoribosyltransferase 1 (HPRT1) was detected by DNA pull‑down assay and Chromatin immunoprecipitation (ChIP) assays. HIF-1α, HPRT1, DNA damage and cell apoptosis related genes were examined by western blot. In addition, RNA sequencing, mass spectrometry and bioinformatics analysis were performed.

**Results:**

We found that mutated EGFR (muEGFR) upregulates HPRT1 to promote purine metabolism and tumorigenesis of EGFR-mutant LUAD. Mechanistically, muEGFR increases HIF-1α expression through protein stability. Meanwhile, up-regulated HIF-1α bound to the promoter of HPRT1 and transcriptionally activates HPRT1 expression, enhancing purine metabolism to maintain rapid tumor cell proliferation in EGFR-mutant LUAD. Further, gefitinib inhibited the synthesis of purine nucleotides, and HPRT1 inhibition increased the sensitivity of gefitinib to EGFR-mutant LUAD.

**Conclusions:**

Our study reveals that muEGFR-HIF-1α-HPRT1 axis plays a key role in EGFR-mutant LUAD and provides a new strategy-inhibiting purine metabolism for treating EGFR-mutant LUAD.

**Supplementary Information:**

The online version contains supplementary material available at 10.1186/s13046-024-03184-8.

## Background

Mutations in the epidermal growth factor receptor (EGFR) are a pivotal determinant in the etiology of lung adenocarcinoma (LUAD) [1]. Currently, EGFR-tyrosine kinase inhibitors (TKIs), such as gefitinib and osimertinib, are the mainstay of first-line therapy for advanced lung cancer with EGFR mutations [2]. Despite their initial high response rates, the development of resistance to these EGFR-TKIs is an unfortunate eventuality that can significantly impair patient outcomes over time [3, 4].

EGFR-mutant LUAD experiences significant alterations in a variety of metabolic pathways, including key metabolic processes such as glycolysis, pentose phosphate pathway, glutathione metabolism and lipid metabolism [5–8]. Metabolic reprogramming, as a defined hallmark of malignant tumor, plays a crucial role in tumor biology [9].Notably, abnormal purine metabolism is intimately linked to tumor progression [10]. Purine nucleotides not only play essential roles in DNA and RNA biosynthesis, but also furnish the necessary energy and cofactor molecules that are critical for sustaining cell survival and driving cell proliferation [11]. Multiple enzymes in purine metabolism are disregulated, which is related to the enhanced proliferation and development of drug resistance in tumor cells [12]. Hypoxanthine phosphoribosyltransferase 1 (HPRT1) is a key enzyme in the salvage pathway of purine nucleotides, facilitating the conversion of hypoxanthine and guanine to their respective mononucleotides [13]. This enzyme is highly expressed in various cancers, and is implicated in the dynamics of cancer progression and the acquisition of drug resistance [14–16]. In small cell lung cancer (SCLC), HPRT1 promotes cell proliferation by enhancing salvage purine synthesis metabolism in glutamine starvation condition [17]. However, the specific mechanism of mutated EGFR (muEGFR) regulated the HPRT1 to reprogram purine metabolism in EGFR-mutant LUAD remains unexplored and has not yet been reported.

In this study, a critical role for HPRT1-mediated purine metabolism in promoting cell proliferation and tumorigenesis in EGFR-mutant LUAD were uncovered. Moreover, the regulatory mechanism and function of hypoxia-inducible factor-1 alpha (HIF-1α) on HPRT1 were clarified. Finally, the effect of targeting purine metabolism on the therapeutic efficacy of EGFR-TKIs was explored.

## Materials and methods

### Cell culture

EGFR wild type lung adenocarcinoma cell (H1299) and EGFR mutant type lung adenocarcinoma cells (PC9, H3255 and H1975) were purchased from the cell bank of the Committee on Type Culture Collection of the Chinese Academy of Sciences (CTCC). H1299, PC9, H3255 and H1975 cell lines were cultured in RPMI 1640 medium (Gibco) supplemented with 10% FBS (Meilun) and 1% penicillin/streptomycin (Meilun) at 37℃ with 5% CO2 in a humidified incubator (Thermo).

## Plasmids construction and viral infection

The HPRT1 overexpression plasmid was constructed in the pCDH-puro vector, and the HPRT1 and HIF-1α short hairpin RNA plasmids were constructed in the pLKO.1-puro backbone. The viruses were produced from 293T cells with lentivirus packaging vectors (PRRE, VSVG and REV) and then infected into target cell lines following manufacturer’s instructions. The sequences of shRNAs are given below:

- shHPRT1#1: CCAGGTTATGACCTTGATTTA
- shHPRT1#2: GCACTGAATAGAAATAGTGAT
- shHIF-1α#1: GTGATGAAAGAATTACCGAAT
- shHIF-1α#2: GCCGCTGGAGACACAATCATA

### RNA extraction and real-time PCR

First, total RNA was extracted with RNAiso Plus reagent (Takara, #9108) following the manufacturer’s instructions. Second, the cDNA was performed using PrimeScript RT reagent kit with gDNA Eraser (TaKaRa, #RR047). Finally, real-time PCR was performed using TB Green Premix Ex Taq II (TaKaRa, #RR036) on PCR machine (LightCycler 96, #Roche). The sequences of primers are as follows:

- GAPDH forward: TCCAAAATCAAGTGGGGCGA
- GAPDH reverse: TGATGACCCTTTTGGCTCCC
- HPRT1 forward: ACAGGACTGAACGTCTTGCT
- HPRT1 reverse: GTCCCCTGTTGACTGGTCATT
- IMPDH1 forward: CGTGCCCTACCTCATAGCAG
- IMPDH1 reverse: GCCGCTTTTCGTAAGAGTGC
- IMPDH2 forward: AGCTCTTCAACTGCGGAGAC
- IMPDH2 reverse: GGATGAAGCCAATACCGCCT
- HIF-1α forward: GGCGCGAACGACAAGAAAAA
- HIF-1α reverse: GTGGCAACTGATGAGCAAGC

### Western blot

Cells were lysed in RIPA lysis buffer containing protease inhibitors. Supernatants were subjected to SDS-PAGE precast glue, and transferred to PVDF membrane (Merck Millipore, #IPVH00005). The membranes were blocked with 5% skim milk powder at 37 ℃ for 1 h, and then incubated with the primary antibodies and second antibodies sequentially. The proteins were visualized with enhanced chemiluminescence (ECL) reagent on Chemiluminescence imaging system (Tanon, #2500). The primary antibodies are as follows: GAPDH (ZEN BioScience, #R24404), HPRT1 (abcom, #ab109021), HIF-1α (Proteintech Technology, #20960-1-AP), EGFR (Cell Signaling Technology, #4267), p-EGFR (abcom, #ab5644), Cleaved Caspase 3 (Cell Signaling Technology, #9661,), p-Chk1 (Proteintech Technology, #28803-1-AP), Chk1 (Proteintech Technology, #25887-1-AP) and γ-H2A.X (abcom, #ab81299).

### Cell proliferation assay

For cell proliferation assay, 1000 cells were seeded in 96-well plates and incubated overnight. Next day, the experiment was carried out in accordance with BeyoClick™ EdU Cell Proliferation Kit with TMB (Beyotime, #C0088).

### Clinical samples

Ten pairs of EGFR wild lung adenocarcinoma cancer tissue and paracancerous tissue samples, as well as nine pairs of EGFR mutant lung adenocarcinoma cancer tissue and paracancerous tissue samples were obtained from the First Affiliated Hospital of Zhengzhou University. All patients provided written informed consent. The study was approved by the ethics committee of the First Affiliated Hospital of Zhengzhou University (2019-ky-30).

### Metabolite extraction

For tissue samples, 20 mg of tissue sample was plated into the EP tube, and then the grinding ball and 500 µL of pre-cooled methanol containing the internal standard solution 1 (Human Metabolome Technologies, #H3304-1002) were added. After ground for 2 min, 500 µL of chloroform was added, vortexing for 30 s. Subsequently, 200 µL of ultrapure water was added, after 30 s of vortex the sample was stood for 10 min, further centrifuged at 4 °C and 13,000 rpm for 15 min, the supernatant was filtered and freeze-dried. Dried metabolite samples were stored at -80 °C.

For cell samples, cells were washed three times with 5% mannitol and then quenched by immersing them in liquid nitrogen. One mL of 100% methanol containing the internal standard solution 1 was added and cells were scraped into the 5 mL EP tube with a scraper. The following process was the same as above.

For isotope-labeled cell samples, cells were treated with 4 mM amide-^15^N-glutamine (Sigma Aldrich, #490024) or 60 µM ^15^N_4_-hypoxanthine (Cambridge Isotope Laboratories, #NLM-8500-PK) for 24 h before collection.

### CE − TOF/MS analysis

The experiment was performed based on a capillary electrophoresis system (CE, #G7100A, Agilent) equipped with a 1260 ISO pump (Agilent, #G1310B) coupled with a time-of-flight mass spectrometry system (TOF/MS, Agilent, #G6224A) with an electrophoresis-electrospray ionization-MS spray kit (Agilent, #G1607A). Agilent’s coaxial sheath fluid interface was used to connect capillary electrophoresis and mass spectrometer. CE and MS are controlled through ChemStation software (Agilent, B.04.03) and Mass Hunter Workstation software (Agilent, B.04.00). Metabolomics data were acquired in both cation-positive (CP) and anion-negative (AN) modes. The original CE-TOF/MS analysis data were processed using the software Qualitative analysis (Agilent, B.04.00), Quantitative Analysis (Agilent, B.04.00), and MethodMarker (Human Metabolome Technologies). Metabolite identification is based on a database constructed from 500 standards. Before statistical analysis, the exported data were sequentially subjected to internal standard normalization.

### RNA sequencing

Total RNA was firstly extracted using a TRIzol total RNA extraction kit (TIANGEN, #DP424), then the library was constructed according to the manufacturer’s instructions and sequenced using the sequencing platform (Illumina, NovaSeq 6000). Gene set enrichment analysis (GSEA) was performed by the function in package clusterProfiler.

### Luciferase reporter assay

293T cells (5 × 10^4^) were seeded in 24-well plates at 5 × 10^4^/well, and then co-transfected with pLenti-EGFR-WT/pLenti-EGFR-Mut (E746-A750del; del19), pGL3.0-HPRT1 promoter and Renilla plasmids with Lipofectamine 2000 (Invitrogen, #11668500) in the next day. pLenti-EGFR-WT (Cat No.: PPL00063-4c) and pLenti-EGFR-Mut (E746-A750del; del19) (Cat No.: PPL00063-4d) plamsids were purchased from Geneppl technology, co, Ltd. pcDNA3.1-HIF-1α, pGL3.0-HPRT1 promoter/pGL3.0-HPRT1 promoter Mut and Renilla plasmids were co-transfected in 293T cells. After 24 h, dual-lucy assay kit (Solarbio, #D0010) was used to examined luciferase activity.

### DNA pull‑down assay

The promoter region of HPRT1 was amplified by PCR using pGL3-Basic HPRT1 promoter as template and 5’-biotin-labeled forward primer. The HPRT1 HRE2 WT promoter sequence and the HPRT1 HRE2 Mut promoter sequence were synthesized by Sangon Biotech (Shanghai) Co., Ltd. The DNA probe was incubated with streptavidin agarose beads (Biovision, #6565-2). Then, the nuclear proteins were extracted from PC9 cells and added to the DNA-beads system and kept on a shaker 4 ℃ overnight. After multiple washes, the bound proteins were collected. Finally, the proteins were used for Coomassie Brilliant Blue staining, MS analysis and western blot. The primers sequences were listed below:

- HPRT1 forward: biotin-GCTGACTGTACTGTCCTAAGTGCAT
- HPRT1 reverse: AGGGCTCGTCGCAGCC
- HPRT1 HRE2 WT: Biotin-AGCCACAGGTAGTGCAAGGTCTTGGGAATGGG**ACGT**CTGGTCCAAGGATTCACGCGATGACTGGAACCCGAA
- HPRT1 HRE2 Mut: Biotin-AGCCACAGGTAGTGCAAGGTCTTGGGAATGGG**AAAA**CTGGTCCAAGGATTCACGCGATGACTGGAACCCGAA

### Chromatin immunoprecipitation (ChIP) assays

Chip was performed using Pierce Agarose Chip Kit (Thermo Scientific, #26156) according to the manufacturer’s protocol. Cells were firstly fixed and crosslinked, then were lysed and digested with Micrococcal Nuclease. Chromatin was immnuoprecipitated with HIF-1α antibody (Cell Signaling Technology, #14179) or IgG antibody. Finally, protein/DNA complexes were eluted, and DNA was released. The later was amplified with the Chip primers. The primers sequences used were listed below:

- HPRT1 HRE1 promoter forward: 5′-AGCCACAGGTAGTGCAAGG-3′
- HPRT1 HRE1 promoter reverse: 5′-TTCGGGTTCCAGTCATCG-3′
- HPRT1 HRE2 promoter forward: 5′-CTTAGAGGCTAGAAGAAA-3′
- HPRT1 HRE2 promoter reverse: 5′-ATAGAGCTTGGCTCAATA-3′
- Positive control (VEGF) forward: AGCAGGAACAAGGGCCTCTGTCT
- Positive control (VEGF) reverse: GGAGGGAAGAGGACCTGTTGGAG
- Negative control forward: TCAGGCTGTGAACCTTGGTGGGG
- Negative control reverse: GCTCTGCGGACGCTCAGTGAAGC

### Animal studies

All animal studies were approved by the Ethics Committee of Dalian Institute of Chemical Physics, Chinese Academy of Sciences (DICPEC2308). 4–6 weeks male nude mice were purchased from Liaoning Changsheng Biotechnology co., Ltd. The cells (5 × 10^6^) were diluted with 100 µL PBS and mixed with 100 µL Matrigel. The suspension was injected subcutaneously into the dorsal flanks of 4–6 weeks male nude mice. Tumor size was measured every three days with a caliper, and the volume was calculated according to the formula: (major axe × minor axe^2^)/2. After the whole experiment, the mice were sacrificed and the tumors were removed and evaluated, followed by immunohistochemistry and metabolomics analysis. To explore whether the inhibition of purine metabolism could enhance gefitinib sensitivity, mice were injected with PC9 cells (5 × 10^6^). When the tumor volume reached 100 mm^3^, the mice were randomly divided into four groups, mice in groups 1, 2, 3 and 4 were treated every day with normal saline, gefitinib (50 mg/kg) by gavage, 6-MP (100 mg/kg) by daily intraperitoneal injection, and the combination of gefitinib and 6-MP, respectively.

### Immunohistochemistry (IHC)

Paraffin sections were dehydrated, and then incubated in 3% H_2_O_2_ to eliminate endogenous peroxidase activity. Sections were blocked with 10% normal goat serum, followed by primary antibodies and biotin-labeled secondary antibodies. The experiment was performed following manufacturer’s instructions (ZSGB-BIO, #PV-9000). HPRT1 (1:150), Ki67 (1:50, Proteintech Technology, #27309-1-AP), HIF-1α (1:100) and γ-H2A.X (1:100) were used as the primary antibodies. H-score was used to assess the results of IHC and calculated according to following equation: H-score = Σpi(i + 1), where pi represents the percentage of positive cells to all cells in the slice, and i represents the staining intensity (0, negative; 1, weak; 2, moderate; and 3, intense).

### Statistical analysis

Data were derived from three or more independent, replicate experiments, and presented as mean ± SD. Student’s t-test (unpaired, two-tailed) with *p* < 0.05 was considered statistically significant between groups. Multiple data analysis was carried out using GraphPad Prism 6 Software (La Jolla, CA, USA), Gene Expression Profiling Interactive Analysis (http://gepia2.cancer-pku.cn/#index), Multi Experiment Viewer (http://www.tm4.org), SIMCA-P Software (Umetrics, Sweden), MetaboAnalyst (http://www.metaboanalyst.ca) and FlowJo 10.6.2 (Becton, Dickinson and Company).

## Results

### Purine metabolism is enhanced in EGFR-mutant LUAD

**Fig. 1:**
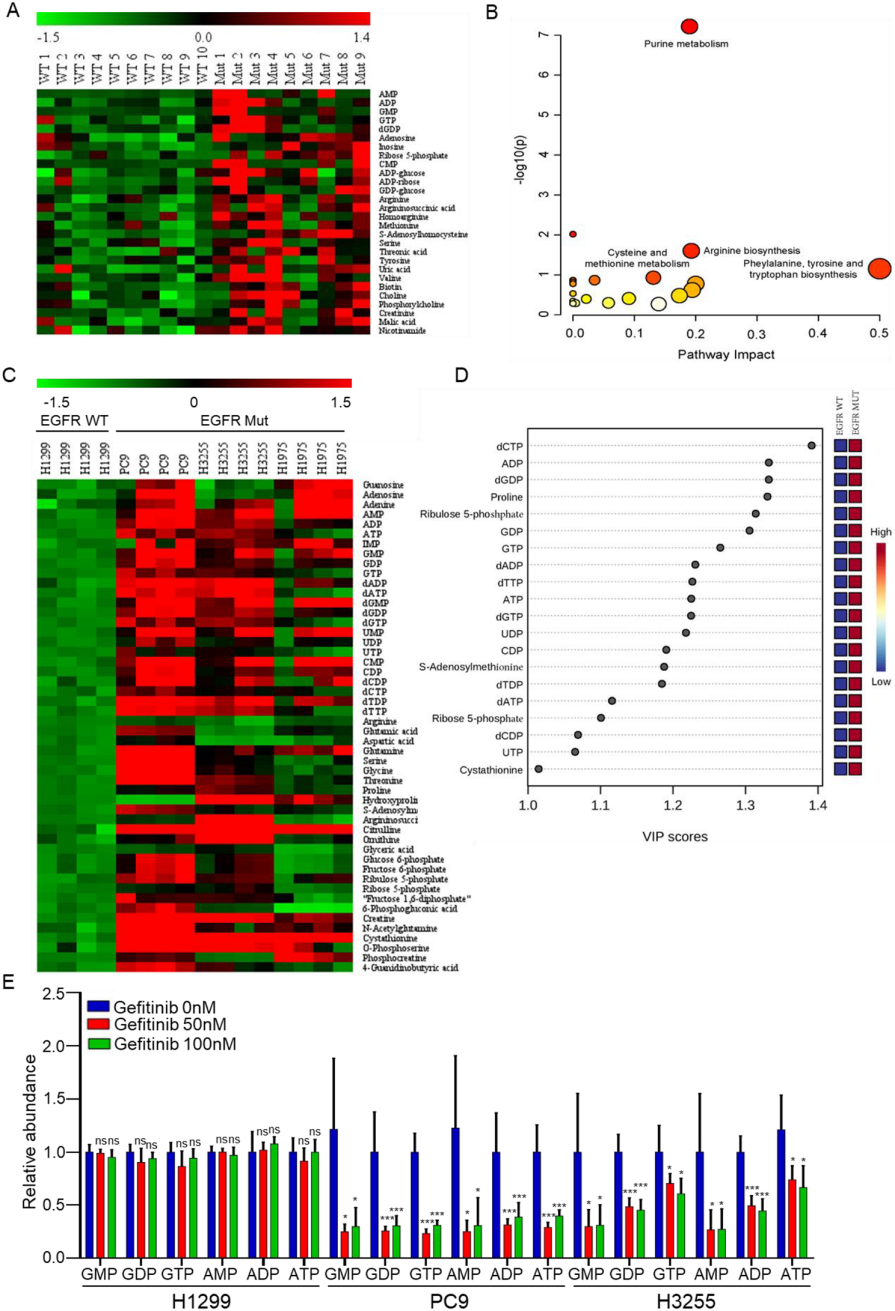
EGFR-mutant LUAD has enhanced purine metabolism. **A** Heatmap analysis of significantly differential metabolites. Metabolomics of tumor tissues and adjacent normal tissues derived from ten pairs of EGFR-WT and nine EGFR-Mut LUAD patients, then the ratio of metabolites in cancerous/adjacent normal tissue of the same patient was used to remove individual differences. *p* < 0.05, unpaired two-tailed Student’s t-test. **B** Pathway enrichment analysis based on significantly differential metabolites. **C** Heatmap analysis of changed metabolites. Metabolites were extracted from EGFR-WT LUAD cells (H1299) and EGFR-Mut LUAD cells (PC9, H3255, H1975). *n* = 4 independent cell cultures for each cell lines. *p* < 0.05, unpaired two-tailed Student’s t-test. **D** Metabolites differentiating EGFR-WT from EGFR-Mut LUAD cell lines had variable importance (VIP scores > 1 were shown). The VIP value reflects the contribution of the metabolite to the classification of the model. Generally, VIP value greater than 1 is considered as a key contributor to the classification of the model. **E** Relative content of metabolites in purine metabolism pathway in H1299, PC9 and H3255 cells without or with gefitinib treatment for 48 h. *n* = 5 independent cell cultures for each group sample. ns, not significant, * *p* < 0.05, *** *p* < 0.001

Rapid proliferating tumors caused by EGFR mutations have abnormally activated purine metabolism [18, 19]. To explore metabolic changes caused by muEGFR in LUAD, 10 pairs of adjacent normal and cancerous tissues from EGFR wild-type (WT) LUAD patients and 9 pairs of tissues from EGFR-mutant (Mut) LUAD patients were subjected to CE-TOF/MS-based metabolomics analysis. Compared with EGFR-WT LUAD tissues, quite a few metabolites altered in EGFR-Mut LUAD tissues (Fig. 1A), significantly changed purine metabolism was found in the pathway enrichment analysis (Fig. 1B). Further, we evaluated the metabolic differences between EGFR-WT and EGFR-Mut LUAD cells, heatmap showed that metabolites in purine metabolism had significant higher levels in EGFR-Mut LUAD cells, than in EGFR-WT LUAD cells (Fig. 1C). Orthognonal partial least squares discriminant analysis (OPLS-DA) showed most of the metabolites had VIP (variable important for the projection) values greater than 1, revealing that metabolites involved in purine metabolism were discriminatory between EGFR-WT and EGFR-Mut LUAD cells (Fig. 1D). To strengthen the verification of the effect of activated EGFR on purine metabolism, H1299, PC9 and H3255 cells were treated with gefitinib (an inhibitor of EGFR-TKIs), the results demonstrated that EGFR inhibition significantly reduced the metabolite contents of the purine metabolism in EGFR-Mut LUAD cells, while no effect was found in EGFR-WT LUAD cells (Fig. 1E). These results suggested that EGFR mutations caused significant purine metabolism reprogramming in EGFR-Mut LUAD.

### Mutated EGFR upregulates HPRT1 expression in EGFR-mutant LUAD

**Fig. 2:**
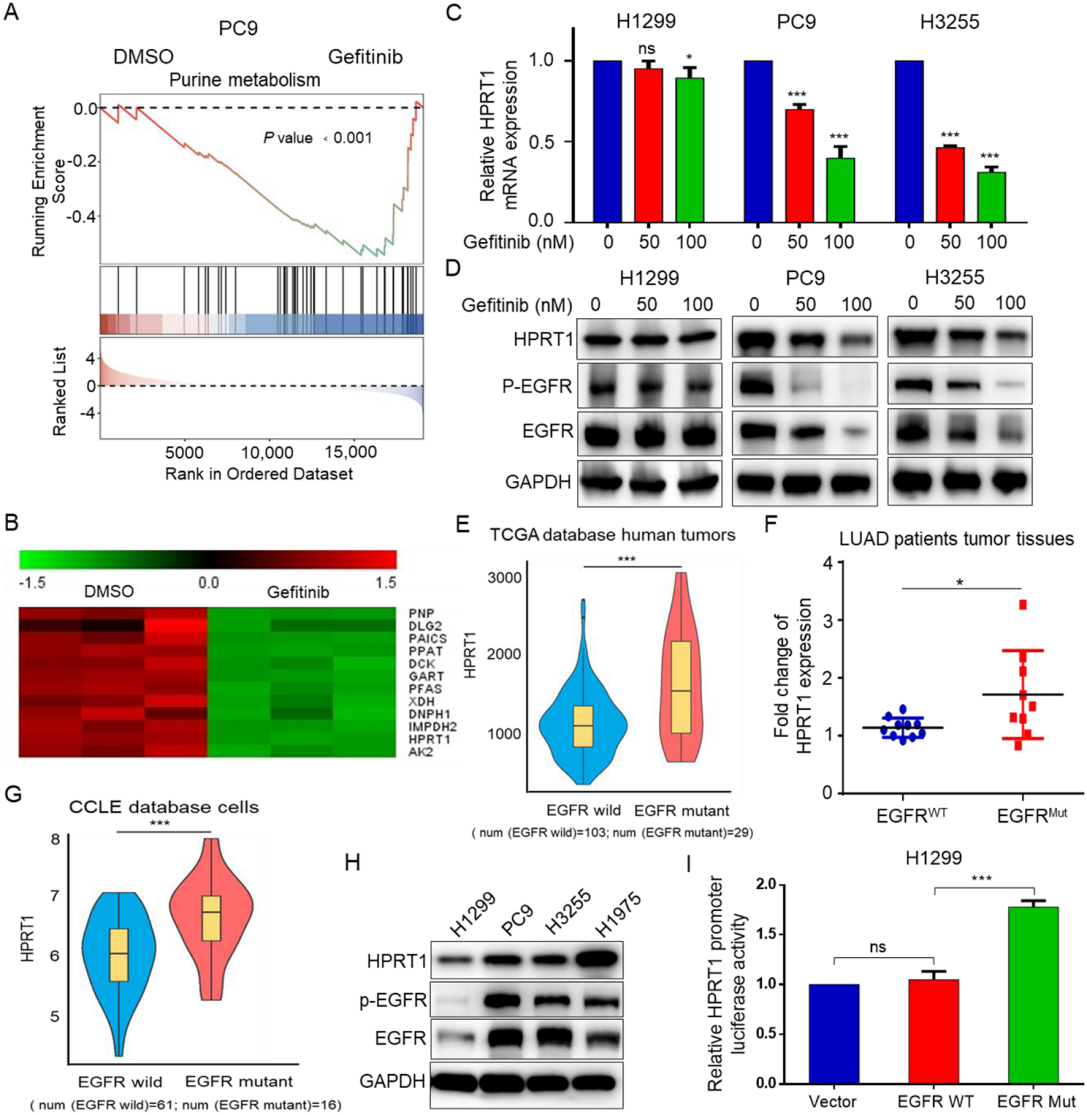
HPRT1 is highly expressed and regulated by EGFR in EGFR-mutant LUAD. **A** Gene set enrichment analysis (GSEA) of purine metabolism gene sets. The data came from RNA sequencing performed in PC9 cells that treated with DMSO or 100 nM gefitinib for 48 h. **B** The heatmap of significantly changed genes of purine metabolism pathway. *p* < 0.05. **C-D** RNA and protein expression of HPRT1 by real-time PCR and western blot. ns, not significant, * *p* < 0.05, *** *p* < 0.001. **E** Expression level of HPRT1 in EGFR-WT LUAD tumors (*n* = 103) and EGFR-Mut LUAD tumors (*n* = 29) based on TCGA dataset. *** *p* < 0.001. **F** Fold change of HPRT1 protein expression in EGFR-WT and EGFR-Mut LUAD patients tumor tissues. * *p* < 0.05. HPRT1 protein expression was shown in Fig. S1A. **G** HPRT1 expression in EGFR-WT LUAD cells (*n* = 61) and EGFR-Mut LUAD cells (*n* = 16) based on CCLE dataset. **H** Protein expression of HPRT1 in EGFR-Mut LUAD cells compared to EGFR-WT LUAD cells by western blot. **I** pLenti-EGFR-WT/pLenti-EGFR-Mut (E746-A750del; del19), pGL3.0-HPRT1 promoter and pRL-TK plasmids were transfected into H1299 cells through lipofectamine 2000 transfection reagent for 48 h, and HPRT1 promoter activity was examined by luciferase reporter assay. ns: no significant; *** *p* < 0.001

To elucidate the metabolic enzymes regulated by muEGFR, PC9 cells were treated with 100 nM gefitinib for 48 h and then subjected to RNA sequencing. Gene set enrichment analysis (GSEA) showed that genes in the purine metabolism pathway were significantly down-regulated (Fig. 2A), the evidently changed genes were presented in Fig. 2B. Real-time PCR and western blot were further carried out, and confirmed that EGFR inhibition reduced the expression of HPRT1 (a key synthetase in the purine salvage synthesis pathway) in EGFR-Mut LUAD cells, while no differential HPRT1 expression was found in EGFR-WT LUAD cells (Fig. 2C, D). Gene expression analysis based on The Cancer Genome Atlas (TCGA) showed that HPRT1 was highly expressed in EGFR-Mut LUAD tissues compared with EGFR-WT LUAD tissues (Fig. 2E).

Subsequently, the expression of HPRT1 in cancerous tissues and the matched adjacent normal tissues from EGFR-WT and EGFR-Mut LUAD patients was examined. The results suggested that HPRT1 expression was higher in EGFR-Mut LUAD tissues (Fig. S1A and Fig. 2F). This discovery was confirmed at the cell line level via transcriptional data analysis obtained from the Cancer Cell Line Encyclopedia (CCLE) (Fig. 2G). Moreover, detection of HPRT1 RNA and protein levels in EGFR-WT and EGFR-Mut cell lines also yielded consistent results (Fig. S1B and Fig. 2H). Further, the effect of muEGFR on HPRT1 promoter activity was assessed by luciferase reporter assay. The results revealed that mutated EGFR, but not wild-type EGFR, enhanced the activity of HPRT1 promoter (Fig. 2I). Taken together, we found that LUAD with EGFR mutations had higher expression of HPRT1 than EGFR-WT LUAD.

### HPRT1 promotes cell proliferation and tumorigenesis by enhancing purine metabolism in EGFR-mutant LUAD

**Fig. 3:**
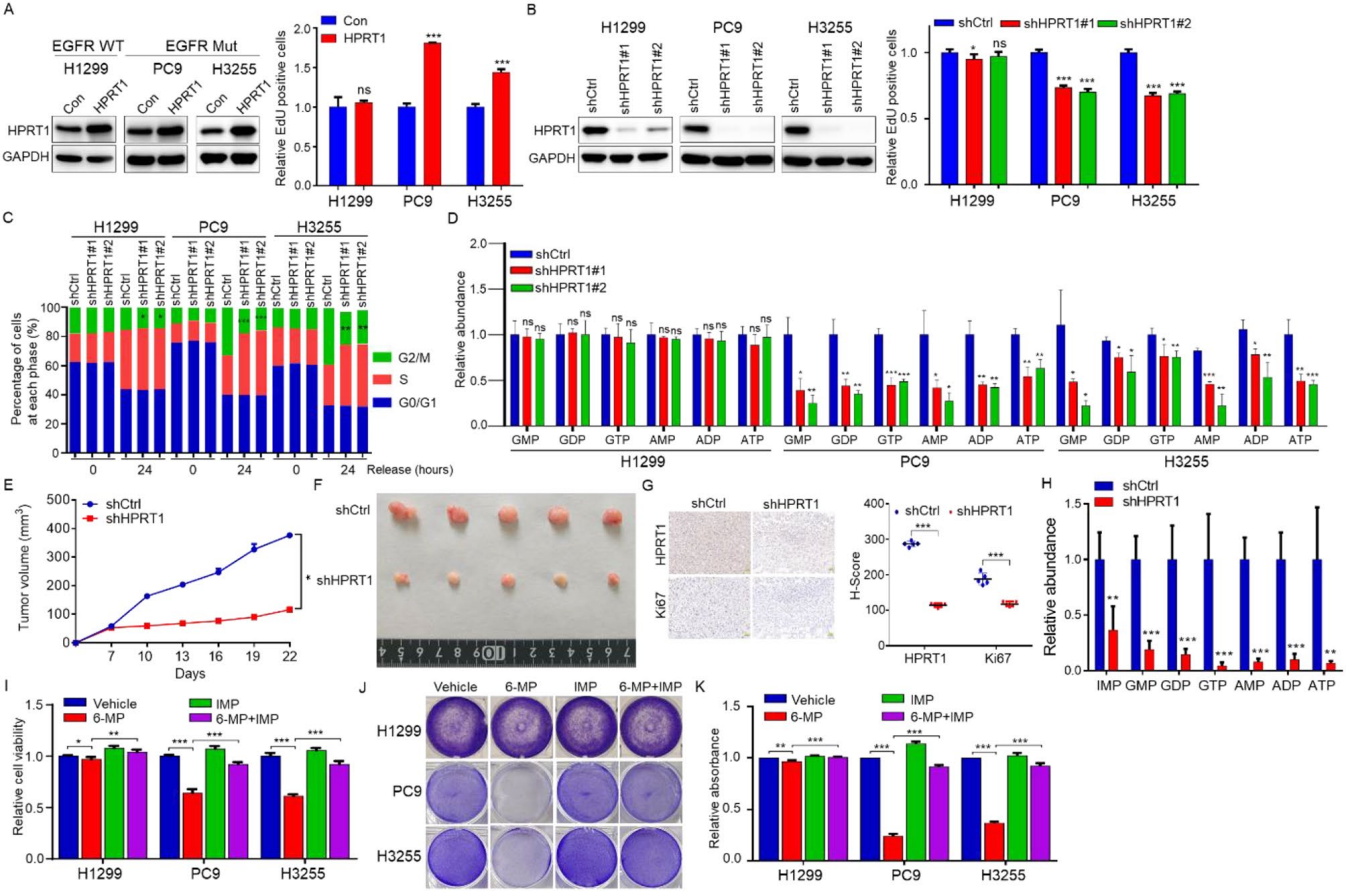
HPRT1 promotes tumorigenesis by increasing the synthesis of purine nucleotides in EGFR-mutant LUAD. **A** The expression of HPRT1 (left), and cell proliferation (right) in HPRT1 overexpressing H1299, PC9 and H3255 cells. ns: not significant, *** *p* < 0.001. **B** Western blot analysis of HPRT1 expression (left) and EdU incorporation assay (right) in HPRT1-silenced (shHPRT1) H1299, PC9 and H3255 cells,. ns: not significant, * *p* < 0.05, *** *p* < 0.001. **C** H1299/PC9/H3255-shHPRT1 and H1299/PC9/H3255-shCtrl cells were serum starved for 24 h, and then returned to normal culture for 24 h for cell cycle assay. *n* = 3 independent replicates. * *p* < 0.05, ** *p* < 0.01, *** *p* < 0.001. **D** Relative content of metabolites in purine metabolism pathway in H1299/PC9/H3255-shHPRT1 cells, H1299/PC9/H3255-shCtrl cells were used as control. ns: not significant, * *p* < 0.05, ** *p* < 0.01, *** *p* < 0.001. **E** Tumor growth monitoration every three days after PC9-shCtrl and PC9-shHPRT1 cells injected subcutaneously into the dorsal flanks of nude mice. * *p* < 0.05. **F** Real photographed tumor images. *n* = 5. **G** Representative images of IHC staining of HPRT1 and Ki67 in xenograft tumor tissues (left), scale bars: 50 μm. H-score was used to assess the results of IHC (right). *** *p* < 0.001. **H** Purine nucleotides measured by CE-TOF/MS in PC9-shCtrl and PC9-shHPRT1 cells-derived tumors. **I** Cell viability assay for various treatment, vehicle, 6-mercaptopurine (6-MP) and IMP alone, 6-MP and IMP combination. * *p* < 0.05, ** *p* < 0.01 and *** *p* < 0.001. **J** Cell growth examination via crystal violet staining. Cells were plated in the 6-well plates, and were treated with vehicle, 6-MP, IMP, 6-MP and IMP combination for 96 h. Representative pictures were presented. **K** Cells from J were added with 33% acetic acid, and then the optical density was measured at 570 nm using a microplate reader. ** *p* < 0.01, *** *p* < 0.001

To evaluate the biological function of HPRT1 in EGFR-mutant LUAD, HPRT1 was overexpressed in H1299, PC9 and H3255 cells. EdU cell proliferation assay demonstrated that HPRT1 overexpression promoted cell proliferation of EGFR-Mut LUAD cells, but not EGFR-WT LUAD cells (Fig. 3A). Furthermore, stable transfected cell lines with HPRT1 knockdown in H1299, PC9 and H3255 cells were constructed. EdU incorporation assay confirmed that cell proliferation was inhibited in HPRT1 knockdown EGFR-Mut LUAD cells, whereas unremarkable cell proliferation change was observed in HPRT1 knockdown EGFR-WT LUAD cells (Fig. 3B). Moreover, cell cycle assay showed that knockdown of HPRT1 prevented the G2/M progression, resulting in reduced cell proliferation ability in PC9 and H3255, however, HPRT1 inhibition had limited effect on H1299 cells (Fig. 3C). HPRT1 overexpression further verified the effect on cell cycle (Fig. S2A). At the metabolic level, we found that the contents of purine nucleotides were dramatically decreased in HPRT1-silenced PC9 and H3255 cells compared with HPRT1-silenced H1299 cells (Fig. 3D). The conclusions drawn from the results of HPRT1 overexpressed cell lines were also consistent (Fig. S2B). Together, these results suggested that HPRT1 was crucial for cell proliferation in EGFR-mutant LUAD cells.

To further explore the effect of HPRT1 on the tumorigenic ability in EGFR-mutant LUAD in vivo, mouse xenograft models were constructed. The results indicated that knockdown of HPRT1 suppressed tumor growth and decreased tumor weight (Fig. 3E, F). IHC assay showed that the expressions of HPRT1 and Ki67 were reduced in tumors formed by HPRT1-silenced cells (Fig. 3G). Meanwhile, CE-TOF/MS-based metabolomics analysis revealed that purine nucleotides obviously decreased in the tumors derived from PC9-shHPRT1 cells compared to PC9-shCtrl cells-derived tumors (Fig. 3H).

Next, to test whether HPRT1-mediated purine metabolism affected cell proliferation, 6-mercaptopurine (6-MP) was used to inhibit the activity of HPRT1 in H1299, PC9 and H3255 cells. Cell viability assay determined that 6-MP treatment decreased cell viability in EGFR-Mut LUAD cells, and IMP addition complemented the ability of cell proliferation, whereas this treatment had a limit effect on EGFR-WT LUAD cells (Fig. 3I). Moreover, crystal violet staining experiment confirmed that the inhibition of cell proliferation caused by 6-MP could be complemented by IMP in EGFR-Mut LUAD cells but not in EGFR-WT LUAD cells (Fig. 3J, K). Taken together, HPRT1 promoted cell proliferation and tumorigenesis through reprogramming purine metabolism in EGFR-mutant LUAD.

### HIF-1α transcriptionally regulates the expression of HPRT1 in EGFR-mutant LUAD

Studies have shown that muEGFR could stabilize the protein expression of HIF-1α in EGFR-mutant NSCLC [20]. In our study, we found that the mRNA and protein expression of HIF-1α was higher in EGFR-mutant LUAD cells than in EGFR-wild LUAD cells (Fig. S3A, B). Gefitinib was used to inhibit the activation of EGFR, real-time PCR and western blot experiments demonstrated that gefitinib impaired the expression of HIF-1α at RNA and protein level in PC9 and H3255 cells (Fig. S3C, D). Further results showed that gefitinib caused a drastic shortened half-life of HIF-1α in PC9 cells with cycloheximide (CHX) treatment, and the effect of gefitinib on HIF-1α was blocked by MG132-proteasome inhibitor (Fig. S3E, F). The results suggested that muEGFR increased the expression of HIF-1α through enhancing the protein stability.

**Fig. 4:**
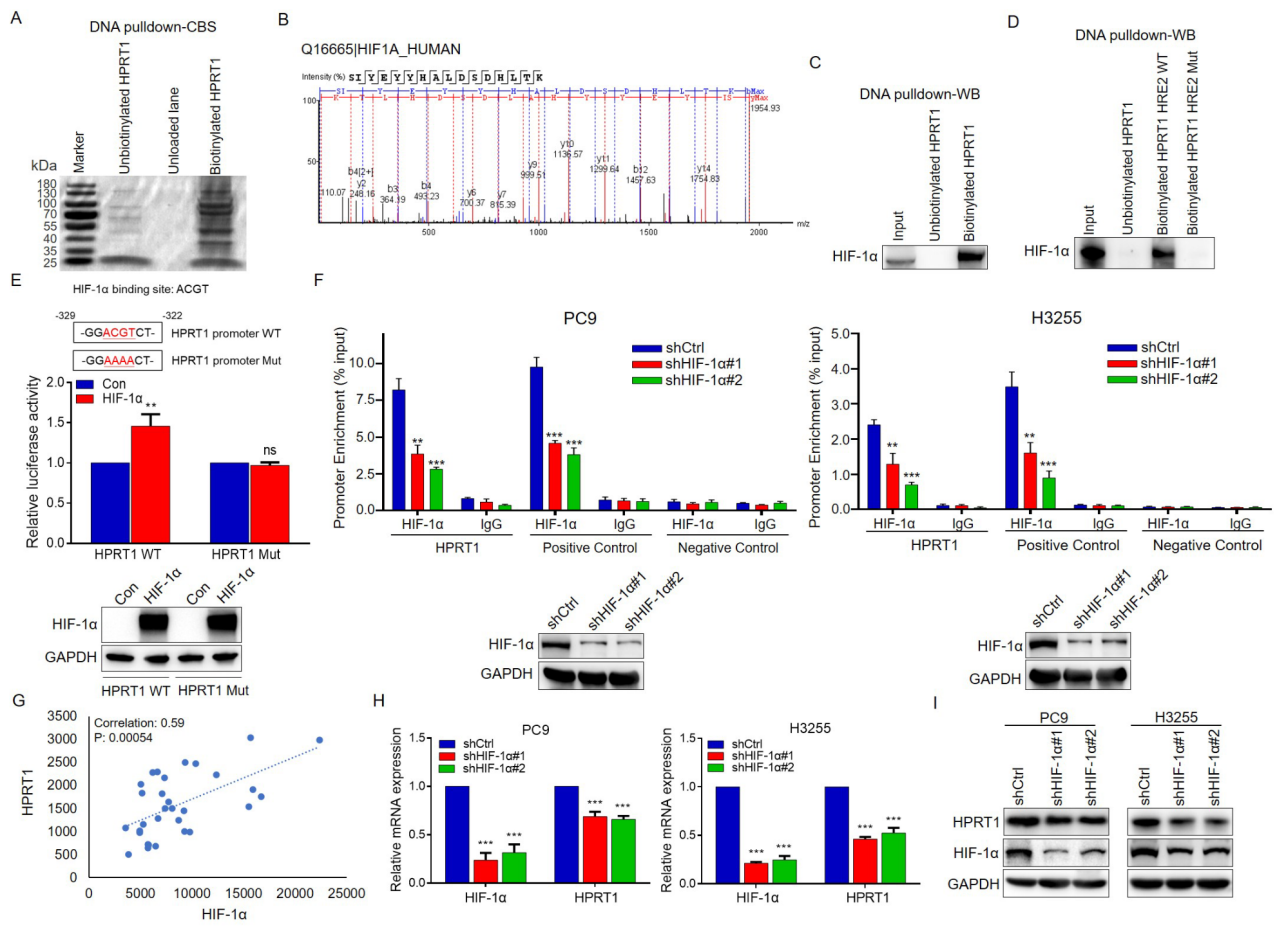
HIF-1α is HPRT1 promoter-binding protein, and regulates the expression of HPRT1. **A** Potential HPRT1 promoter-binding proteins after SDS-PAGE and Coomassie brilliant blue staining. **B** The binding proteins identified by mass spectrometry analysis. **C** The combination of HIF-1α and HPRT1 promoter verified by western blot. **D** DNA pulldown experiment performed with biotinylated HPRT1 HRE2 WT and Mut probes in PC9 cells. **E** Luciferase reporter assay performed in 293T cells co-transtected with HPRT1 promoter WT, HPRT1 promoter mut, HIF-1α plasmids (up). ns, not significant, ** *p* < 0.01. The expression of HIF-1α examined by western blot (down). **F** The level of HIF-1α presence at the promoter of HPRT1 via qChIP assay. VEGF was used as a positive control, and irrelevant sequence was used as a negative control (up). ** *p* < 0.01. The expression of HIF-1α examined by western blot (down). **G** The correlation analysis between HIF-1α and HPRT1 in EGFR-mutant LUAD patients based on TCGA database. **H-I** The expression of HPRT1 in PC9-shHIF-1α and H3255-shHIF-1α cells by real-time PCR and western blot experiments

To explore the upstream key factors that regulated HPRT1 expression in EGFR-mutant LUAD, biotinylated primer was designed and synthesized based on the HPRT1 promoter sequence, and biotinylated DNA probe was obtained by PCR amplification. Proteins bound to the HPRT1 promoter was captured using DNA pull down assay. Coomassie brilliant blue staining and mass spectrometry identified that the transcription factor HIF-1α might bind to the promoter of HPRT1 (Fig. 4A, B). Furthermore, we verified the binding of HIF-1α to the HPRT1 promoter by western blot experiment (Fig. 4C). Subsequently, The Eukaryotic Promoter Database (EPD) was used to search for potential HIF-1α binding sites on the HPRT1 promoter. We found that the HPRT1 promoter contained two potential HREs (HRE1, which was located at -1947–1940 bp, and HRE2, which was located at -329–322 bp). ChIP assay with HIF-1α antibody confirmed that HIF-1α could bind to the region containing HRE2 (Fig. S4). To further verify this result, biotin-labeled HRE2 WT and Mut probes were used to perform DNA pull down experiment, and results demonstrated that HRE2 was the binding site of HIF-1α (Fig. 4D). To confirm whether HIF-1α transcriptionally regulated HPRT1, luciferase reporter assay was performed in 293T cells. The results showed that HIF-1α enhanced wild-type HPRT1 promoter activity, whereas it had no effect on the mutated HPRT1 promoter activity (Fig. 4E). Furthermore, HIF-1α was knocked down by two small-hairpin (shRNA) in PC9 cells and H3255 cells. We found that HIF-1α knockdown led to obvious reduction in the binding of HIF-1α to HPRT1 promoter by ChIP assay (Fig. 4F). Next, analysis based on the TCGA database showed a positive correlation between HIF-1α and HPRT1 in EGFR-mutant LUAD tissues (Fig. 4G). At the cellular level, HPRT1 was significantly decreased in HIF-1α-silenced PC9 and H3255 cells (Fig. 4H, I). Our results confirmed that the expression of HPRT1 was regulated by HIF-1α at the transcriptional level.

#### HIF-1α promotes HPRT1-mediated purine metabolism and tumorigenesis

**Fig. 5:**
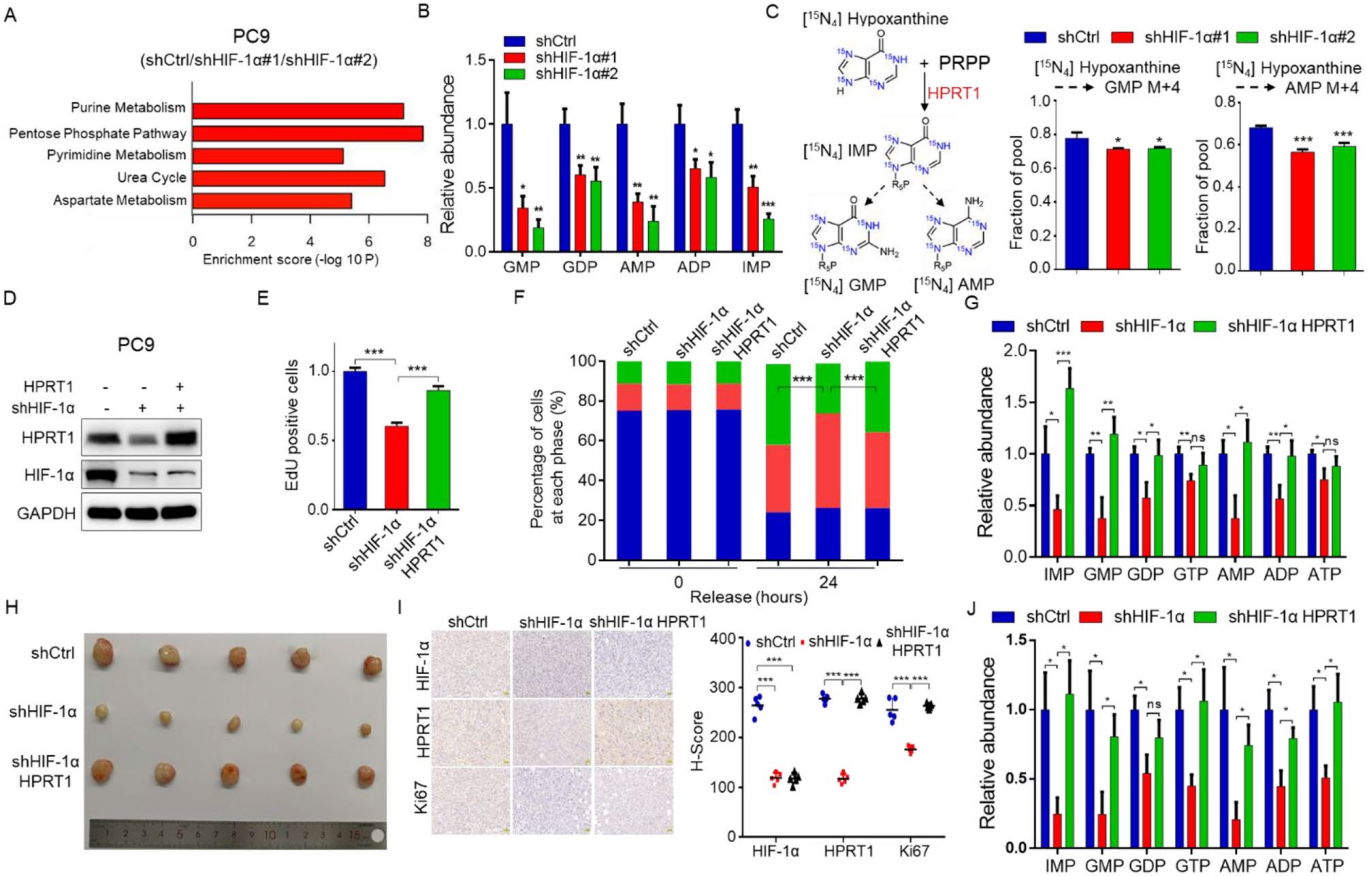
HIF-1α upregulates HPRT1 to promote purine metabolism and tumorigenesis in EGFR-mutant LUAD. **A-B** Differential metabolites in PC9-shCtrl and PC9-shHIF-1α cells detected by CE-TOF/MS based metabolomics. Enrichment analysis based on different metabolites (**A**), relative abundance of purine metabolism intermediates in PC9-shCtrl and PC9-shHIF-1α cells (**B**). **C** Schematic illustrating ^15^N_4_ labeling of nucleotides from ^15^N_4_-hypoxanthine (left), and ^15^N_4_-hypoxanthine-labeled purine metabolism intermediates in PC9-shCtrl and PC9-shHIF-1α cells measured by CE-TOF/MS based metabolomics (right). Data were assessed by two-tailed Student’s t-test. * *p* < 0.05, *** *p* < 0.001. **D** The expression of HIF-1α, HPRT1 analyzed by western blot in PC9-shCtrl, PC9-shHIF-1α and PC9-shHIF-1α HPRT1 cells. **E-F** EdU incorporation assay (E) and cell cycle assay (F) performed in PC9-shHIF-1α and PC9-shHIF-1α HPRT1 cells, PC9-shCtrl cells was used as control. **G** Relative content of IMP, GMP, GDP, GTP, AMP, ADP and ATP evaluated based on CE-TOF/MS metabolomic analysis in PC9-shCtrl, PC9-shHIF-1α and PC9-shHIF-1α HPRT1 cells. **H** Tumor images of Balb/c nude mice after 23 days’ injection with PC9-shCtrl, PC9-shHIF-1α and PC9-shHIF-1α HPRT1 cells. **I** IHC staining of HIF-1α, HPRT1 and Ki67 in tumor tissues. The representative images were shown. Scale bars: 50 μm (left). The H-score of IHC results (right). *** *p* < 0.001. **J** The analysis of purine nucleotides content derived from tumor tissues. ns, not significant, * *p* < 0.05

To elucidate the impact of HIF-1α on HPRT1 function in EGFR-mutant LUAD, we first examined the changes in HIF-1α-mediated metabolic reprogramming. Metabolites levels of PC9-shCtrl and PC9-shHIF-1α cells were analyzed using CE-TOF/MS. Pathway enrichment analysis showed the altered purine metabolism (Fig. 5A). The contents of metabolites in the purine metabolism were significantly decreased in HIF-1α-silenced PC9 cells (Fig. 5B). Further, a stable isotope-labeled metabolic flux technique was used to examine the effect of HIF-1α knockdown on purine nucleotide synthesis. Isotope labeling experiment using amide-^15^N-glutamine showed that HIF-1α knockdown didn’t alter de novo purine metabolism (Fig. S5A). However, using ^15^N_4_-hypoxanthine for isotope labeling experiment, we found that HIF-1α knockdown reduced the synthesis rate of purine metabolism from the salvage synthesis pathway (Fig. 5C). Taken together, the results indicated that HIF-1α regulated HPRT1-mediated purine salvage synthesis pathway.

Next, HPRT1 was overexpressed in HIF-1α-silenced PC9 cells (Fig. S5B and Fig. 5D). The EdU incorporation assay demonstrated that the ability of cell proliferation was dramatically reduced by HIF-1α knockdown, and was restored when HPRT1 was overexpressed in PC9-shHIF-1α cells (Fig. 5E). Cell cycle assay showed that the proportion of cells in the G2/M phase was severely reduced in PC9-shHIF-1α cells, and the effect was reversed by HPRT1 overexpression (Fig. 5F). To verify whether HIF-1α-HPRT1 axis promoted purine metabolism, CE-TOF/MS-based metabolomics analysis was performed. The results revealed that the contents of purine nucleotides, such as AMP, GMP and IMP, were restored in HPRT1 overexpressed PC9-shHIF-1α cells (Fig. 5G).

Finally, to examine the effect of the HIF-1α-HPRT1 axis on tumorigenesis in vivo, PC9-shCtrl, PC9-shHIF-1α and PC9-shHIF-1α HPRT1 cells were subcutaneously injected into the dorsal flank of mice to construct mouse xenograft models. The results showed that HIF-1α knockdown inhibited tumor growth, while HPRT1 overexpression restored it (Fig. 5H). The expression of HIF-1α, HPRT1 and Ki67 in mouse tumor tissues was detected by IHC (Fig. 5I). Moreover, the contents of purine nucleotides were decreased in PC9-shHIF-1α cells-derived tumor tissues compared to control group, while the overexpression of HPRT1 restored the contents of purine nucleotides (Fig. 5J).

### Inhibition of HPRT1 sensitizes EGFR-mutant LUAD to EGFR-TKI

**Fig. 6:**
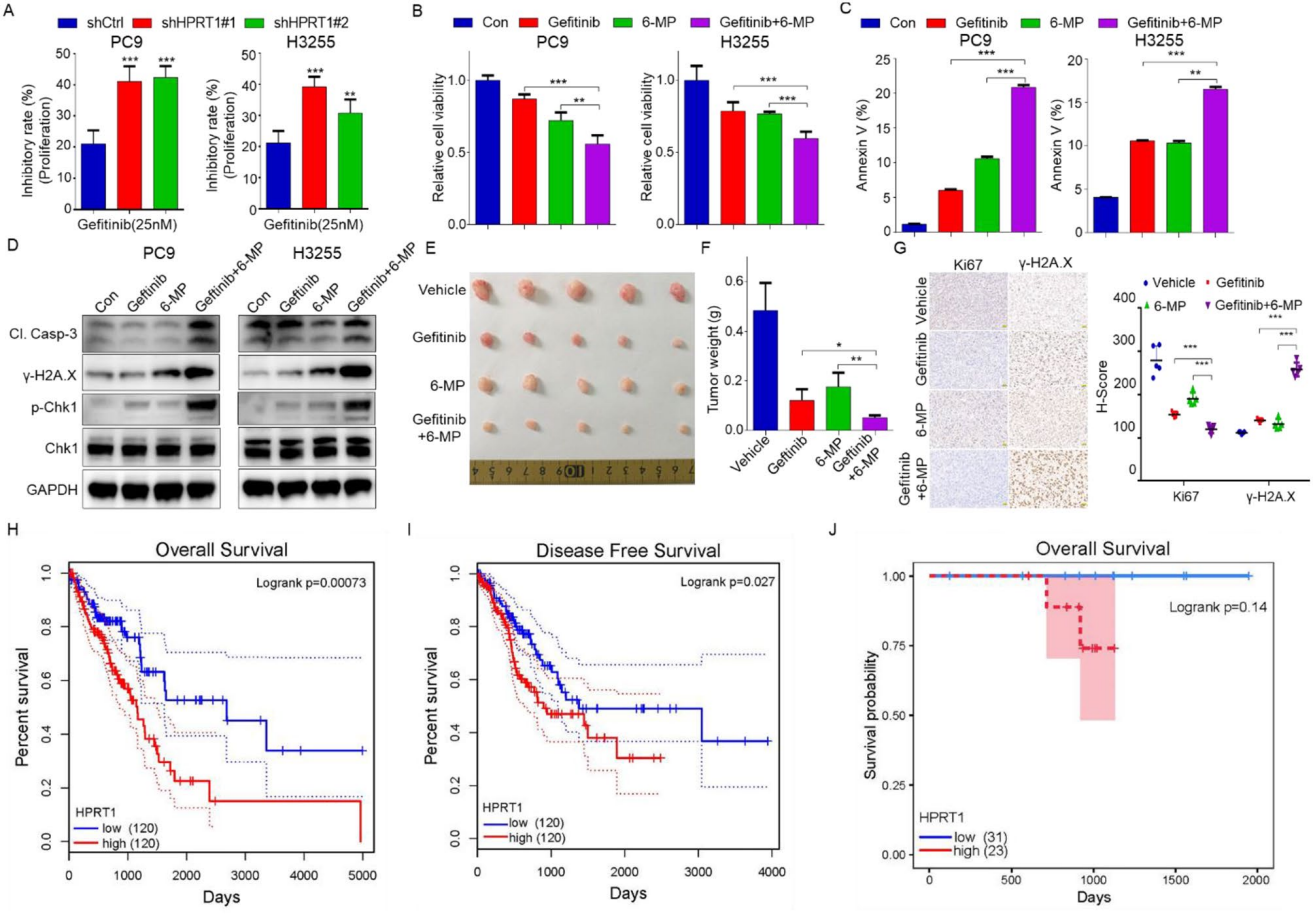
HPRT1 inhibition enhances the sensitivity of EGFR-mutant LUAD cells to EGFR-TKI. **A** Cell viability assay performed in PC9-shCtrl and PC9-shHPRT1 cells with 25 nM gefitinib treatment for 48 h, and the inhibition rate of proliferation was calculated. ** *p* < 0.01, *** *p* < 0.001. **B** Cell viability of PC9 cells treated with 6-MP and gefitinib alone or combination for 48 h. ** *p* < 0.01, *** *p* < 0.001. **C** Cell apoptosis analyzed by PI/Annexin V staining of PC9 cells, which subjected to the same treatment with that in Fig B,. The positive proportion of annexin V was calculated. ** *p* < 0.01, *** *p* < 0.001. *n* = 3 independent replicates. **D** Protein expression of Cleaved Caspase 3 (Cl. Casp-3), γ-H2A.X and p-Chk1 examined by western blot in PC9 cells, which subjected to the same treatment with that in Fig B. **E** Real photographed tumor images. After the formation of PC9 cells derived xenograft tumors, mice were treated with 6-MP and gefitinib alone or combination every day. After 18 days of administration, the tumors were removed. **F** Tumor weighs were calculated. * *p* < 0.05, ** *p* < 0. 01. **G** Representative images of IHC staining of Ki67 and γ-H2A.X in xenograft tumor tissues, scale bars: 50 μm (left). H-score is presented to assess the results of IHC (right). *** *p* < 0.001. **H-I** The overall survival and disease free survival of patients expressing low or high HPRT1 in LUAD based on GEPIA2. **J** The overall survival of HPRT1 in EGFR-mutant LUAD based on GSE72094 datasets

Gefitinib was one of the first-line routine drugs for the treatment of advanced EGFR-mutant LUAD, whether HPRT1 promoted resistance to EGFR-targeted therapy was unclear. In our study, PC9-shCtrl and PC9-shHPRT1 cells were treated without or with gefitinib, cell viability assay indicated that the inhibitory rate of cell proliferation was more significant in PC9-shHPRT1 cells than in PC9-shCtrl cells (Fig. 6A). Meantime, PC9 cells co-treated with 6-MP and gefitinib showed lower cell viability than other groups (Fig. 6B). Cell apoptosis assay by flow cytometry demonstrated that PC9 cells treated with the combination of 6-MP and gefitinib appeared to have the strongest apoptosis (Fig. 6C). Furthermore, the western blot experiment revealed that the combination of 6-MP and gefitinib induced cell apoptosis and DNA damage (Fig. 6D). These results demonstrated that the inhibition of HPRT1 increased the sensitivity of gefitinib to EGFR-mutant LUAD cells.

To further study the effects in vivo, PC9 cells were injected subcutaneously into male balb/c nude mice, then mice were treated with gefitinib and 6-MP alone or in combination. The results showed that mice with the treatment of gefitinib and 6-MP combination had much smaller tumor volumes and weights than in other groups (Fig. 6E, F). Moreover, IHC staining with Ki67 and γ-H2A.X antibodies revealed that 6-MP combined with gefitinib inhibited tumor growth (Fig. 6G).

In summary, the inhibition of HPRT1 made tumors more sensitive to gefitinib treatment. Finally, the correlation of HPRT1 with the prognosis of LUAD was evaluated using the GEPIA2 database. The overall survival and disease free survival cures revealed that highly expressed HPRT1 was positively associated with poor prognosis in LUAD (Fig. 6H, I). Furthermore, based on prognostic information of EGFR-mutant LUAD patients in GSE72094, highly expressed HPRT1 showed a trend toward poor prognosis but there was no significant difference (Fig. 6J).

## Discussion

In this study, we observed that purine metabolism was enhanced in EGFR-mutant LUAD cells and patient tissues. Purine metabolism is recognized as a significant contributor to cellular growth and proliferation. In tumor cells, purine metabolism usually occurs abnormally, resulting in an increase or decrease in the contents of intracellular purine nucleotides, which in turn affects the proliferation and drug resistance of tumor cells [21]. Purine-metabolizing enzymes can disrupt the balance of purine pools, thereby interfering with proliferation and migration of tumor cells. The enzymes involved in purine metabolism are overexpressed in various cancer, such as adenosine deaminase (ADA), cytoplasmic-5’-nucleotidase-II (CN-II) and inosine monophosphate dehydrogenase (IMPDH1) [22–24]. In this study, we found that HPRT1 was overexpressed in EGFR-mutant LUAD cells and tissues, and HPRT1 promoted cell proliferation in vitro and tumor growth in vivo. Similarly, Wang et al. demonstrated that the overexpression of HPRT1 promotes proliferation and migration in head and neck squamous cell carcinoma [25]. HPRT1 has also been found to promote cisplatin resistance by activating phosphoinositide 3-kinase (PI3K)/AKT pathway in oral squamous cell carcinoma [16]. Furthermore, salvage purine synthesis pathway mediated by HPRT1 supports cell growth in SCLC [17, 26]. Our results are consistent with theirs [17, 26], we found that HPRT1-mediated purine metabolism was essential for cell proliferation of EGFR-mutant LUAD. However, our results confirmed that HPRT1 had no effect on the proliferation and purine metabolism of EGFR-WT LUAD cells, possibly because other enzymes in purine metabolism or other pathways antagonize the role of HPRT1 in EGFR-WT LUAD. Similarly, Li et al. reported that PAICS (a de novo purine metabolic enzyme) knockdown could reduce the cell viability of EGFR-WT cells, but had no effect on EGFR-Mut cells. Moreover, after the PAICS gene was knocked out, the p-AKT protein level in EGFR-Mut NSCLC cells was significantly higher than that in EGFR-WT NSCLC cells. Furthermore, BKM120 (a pan-PI3K inhibitor) treatment resulted in a significant decrease in the proliferative capacity in PAICS knockdown EGFR-Mut NSCLC cells, suggesting that activated PI3K-AKT signaling may impair the effects of PAICS knockdown [27].

MuEGFR upregulates HIF-1α expression by stabilizing HIF-1α protein in a hypoxia-independent manner [20, 28]. Consistently, our study verified that the upregulation of HIF-1α was regulated by muEGFR by enhancing protein stability of HIF-1α. HIF-1α acts a transcription factor to regulate the transcription of multiple metabolic enzymes, thereby causing tumor metabolic reprogramming. Specifically, HIF-1α enhances glycolysis and PPP by regulating the expression of pyruvate kinase M2 (PKM2) and glucose-6-phosphate dehydrogenase (G6PD) to impart 5-fluorouracil resistance in colorectal cancer [29]. HIF-1 drives lipid deposition by suppressing carnitine palmitoyltransferase 1 A (CPT1A) to promote tumor growth in clear cell renal cell carcinoma [30]. We found that HIF-1α directly bound to the promoter of HPRT1, and transcriptionally activated the expression of HPRT1 to promote purine nucleotides synthesis in EGFR-mutant LUAD cells.

6-MP is an HPRT1 inhibitor and has been widely used clinically as an antileukemia drug and an immunosuppressive drug [31, 32]. In this study, 6-MP combined with gefitinib was used to study the effect on tumor growth of EGFR-mutant LUAD. The results revealed that EGFR-mutant LUAD cells treated with HPRT1 inhibitor were more sensitive to gefitinib in vitro and in vivo. Wang et al. demonstrate that the inhibition of HPRT1 increases the anticancer effect of EGFR-TKI in head and neck squamous cell carcinoma [25]. In addition, combination treatment of 6-MP and temozolomide has been found to inhibit brain tumor growth [33]. 6-MP combined with methotrexate and methionine sulfoximine suppress the tumor growth of SCLC [17]. Whether 6-MP can increase the effectiveness of EGFR-TKIs-resistant LUAD patients and prolong the survival of patients requires further in-depth study.

## Conclusions

**Fig. 7:**
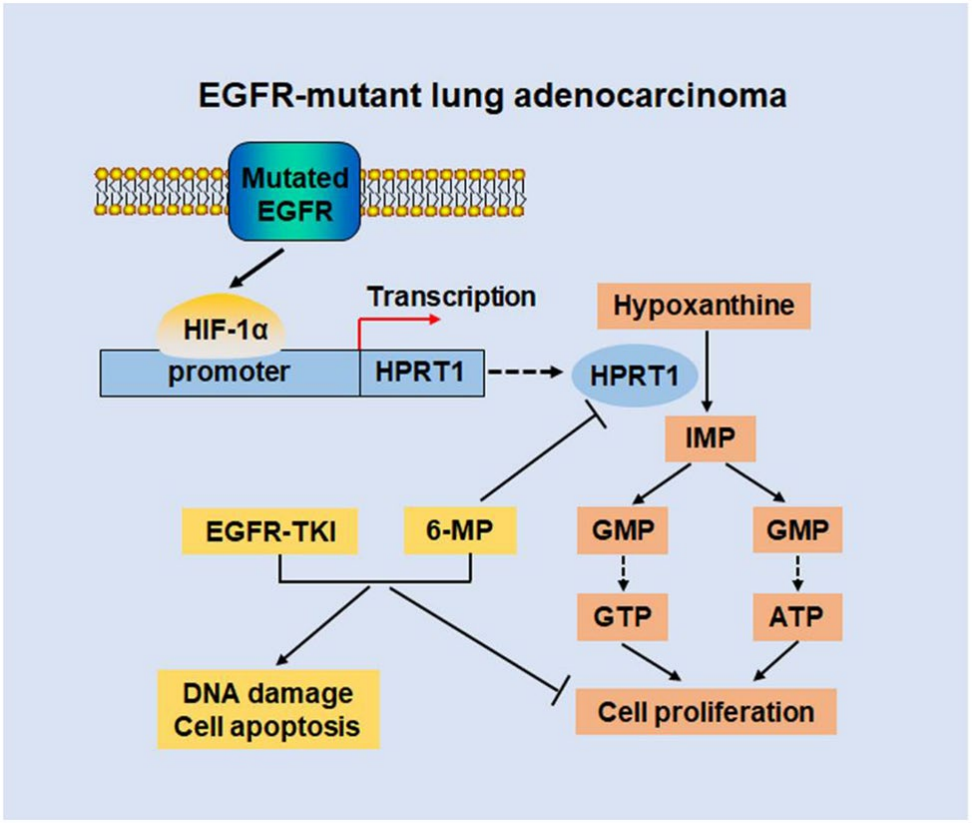
Schematic diagram of muEGFR-HIF-1α-HPRT1 function axis. In EGFR-mutant lung adenocarcinoma, mutated EGFR upregulates HIF-1α expression through protein stability. Furthermore, HIF-1α binds to the promoter of HPRT1 and promotes its transcription. Upregulated HPRT1 enhances purine metabolism to promote purine nucleotide synthesis, ultimately accelerating cell proliferation. Finally, 6-MP combined with EGFR-TKI inhibits cell proliferation and induces DNA damage and cell apoptosis

Our study reveals disorders of purine metabolism in EGFR-mutant LUAD from the perspective of metabolism reprogramming. It is confirmed that purine metabolism catalyzed by HPRT1 promotes the proliferation of EGFR-mutant LUAD in vitro and in vivo. Furthermore, the study of the mechanism shows that HIF-1α transcriptionally regulates HPRT1 to accelerate purine nucleotides synthesis to promote cell proliferation and tumorigenesis. Finally, inhibition of HPRT1 coupled with EGFR-TKIs significantly inhibits the tumor growth of EGFR-mutant LUAD (Fig. 7). The study indicates that intervening purine metabolism of tumors may be a new target for clinical treatment of EGFR-mutant LUAD.

## Electronic supplementary material

Below is the link to the electronic supplementary material.

Supplementary Material 1

## Data Availability

The authors declare that all data supporting the findings of this study are available in this article and its supplementary files.

## Abbreviations

EGFR: Epidermal growth factor receptor
LUAD: Lung adenocarcinoma
HPRT1: Hypoxanthine phosphoribosyltransferase 1
HIF-1α: Hypoxia-inducible factor-1 alpha
TKIs: Tyrosine kinase inhibitors
SCLC: Small cell lung cancer
GSEA: Gene set enrichment analysis
TCGA: The Cancer Genome Atlas
CCLE: Cancer Cell Line Encyclopedia
6-MP: 6-mercaptopurine
EPD: Eukaryotic Promoter Database
ADA: Adenosine deaminase
CN-II: Cytoplasmic-5’-nucleotidase-II
IMPDH1: Inosine monophosphate dehydrogenase
PI3K: Phosphoinositide 3-kinase
PKM2: Pyruvate kinase M2
G6PD: Glucose-6-phosphate dehydrogenase
CPT1A: Carnitine palmitoyltransferase 1 A
ChIP: Chromatin immunoprecipitation
IHC: Immunohistochemistry
