# Supplementary material for: HIF-1α-HPRT1 axis promotes tumorigenesis and gefitinib resistance by enhancing purine metabolism in EGFR-mutant lung adenocarcinoma
Source: J Exp Clin Cancer Res. 2024 Sep 30;43:269. doi: 10.1186/s13046-024-03184-8 (PMC11441087; doi:10.1186/s13046-024-03184-8)
Supplement: Supplementary file 1 — Supplementary Material 1 [file 13046_2024_3184_MOESM1_ESM.docx]

**Supplementary Information**

**HIF-1α-HPRT1 axis promotes tumorigenesis and gefitinib resistance by enhancing purine metabolism in EGFR-mutant lung adenocarcinoma**

**Authors**

Pengyu Geng^1,2^, Fei Ye^3^, Peng Dou^1,2^, Chunxiu Hu^1,2^, Jiarui He^4^, Jinhui Zhao^1,2^, Qi Li^1,2^, Miao Bao^5^, Xiangnan Li^3^*, Xinyu Liu^1,2^* and Guowang Xu^1,2^*

^1^State Key Laboratory of Medical Proteomics, CAS Key Laboratory of Separation Science for Analytical Chemistry, Dalian Institute of Chemical Physics, Chinese Academy of Sciences, Dalian, 116023, Liaoning Province, China

^2^Liaoning Province Key Laboratory of Metabolomics, Dalian, 116023, Liaoning Province, China

^3^The First Affiliated Hospital of Zhengzhou University, Zhengzhou, 450052, Henan Province, China

^4^Clinical Laboratory, The Second Hospital of Dalian Medical University, Dalian, 116023, Liaoning Province, China

^5^Department of Medical Oncology, The First Affiliated Hospital of Xi'an Medical University, Xi'an, 710082, Shanxi Province, China

*Correspondences: Guowang Xu ([xugw@dicp.ac.cn](mailto:xugw@dicp.ac.cn)); Xinyu Liu ([liuxy2012@dicp.ac.cn](mailto:liuxy2012@dicp.ac.cn)); Xiangnna Li (lxn-2000@163.com)


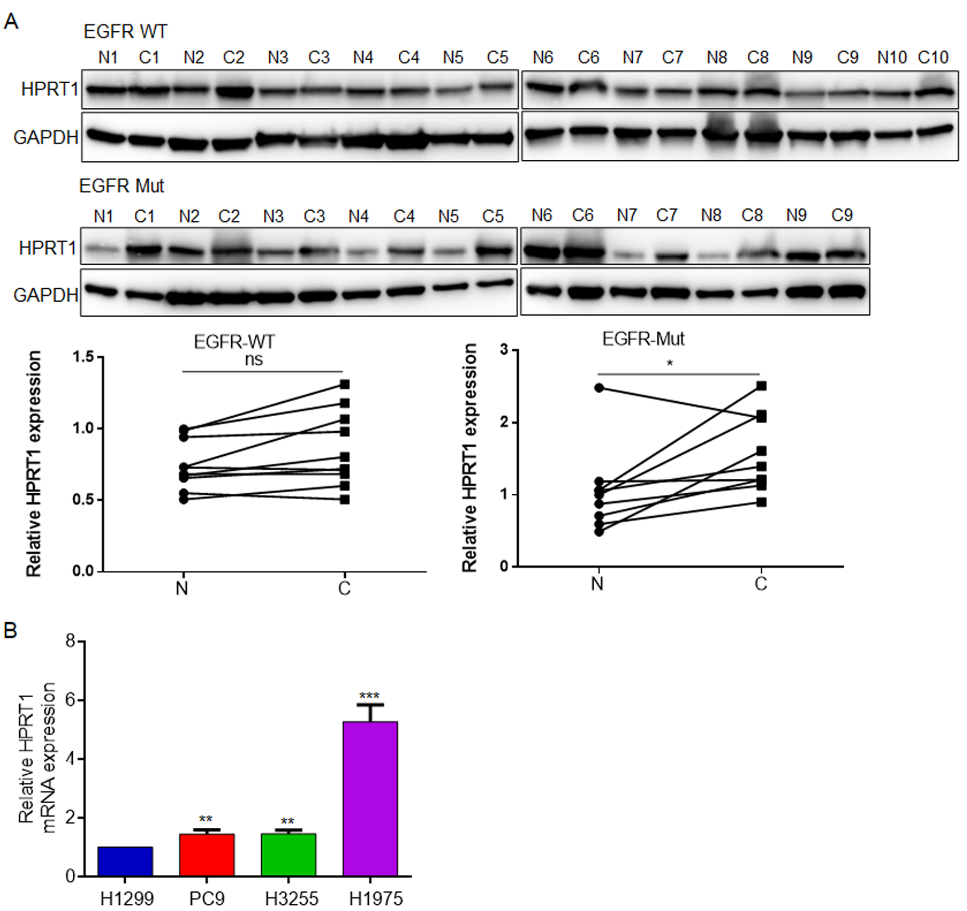


**Fig. S1** The expression of HPRT1 in EGFR-mutant LUAD. **A** Protein expression of HPRT1 in EGFR-WT and EGFR-Mut LUAD patients’ cancerous (C) tissues and adjacent normal (N) tissues. Image J was used for quantitative analysis. **B** mRNA expression of HPRT1 in H1299, PC9, H3255 and H1975 cells. * *p* < 0.05. ** *p* < 0. 01, *** *p* < 0.001.


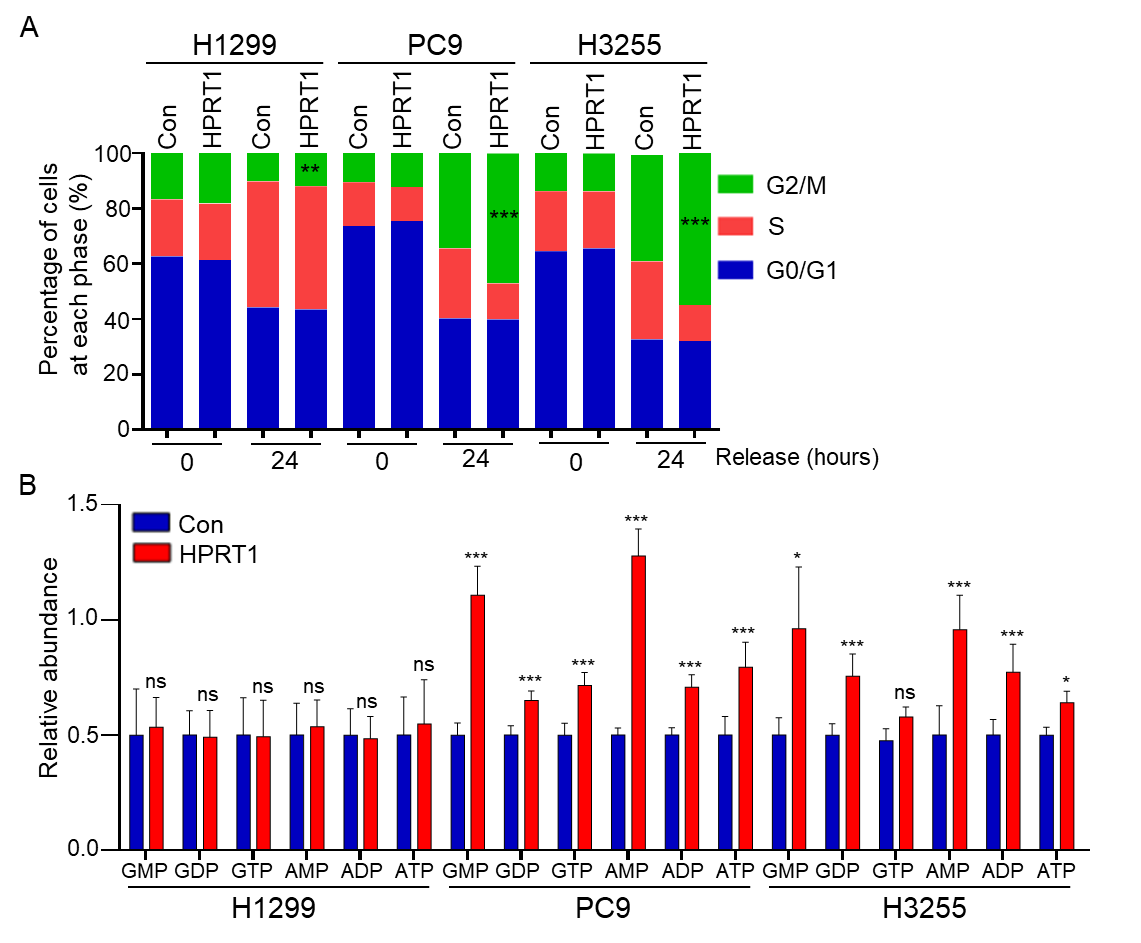


**Fig. S2** HPRT1 promotes cell proliferation and the synthesis of purine nucleotide. A cell cycle assay of H1299/PC9/H3255-Con and H1299/PC9/H3255-HPRT1 cells in the condition of serum starved for 24 h, and then returned to normal culture for 24 h . n=3 independent replicates. B Relative content of purine nucleotide acquired by CE-TOF/MS. ns, not significant, * *p* < 0.05, ** *p* < 0.01, *** *p* < 0.001.


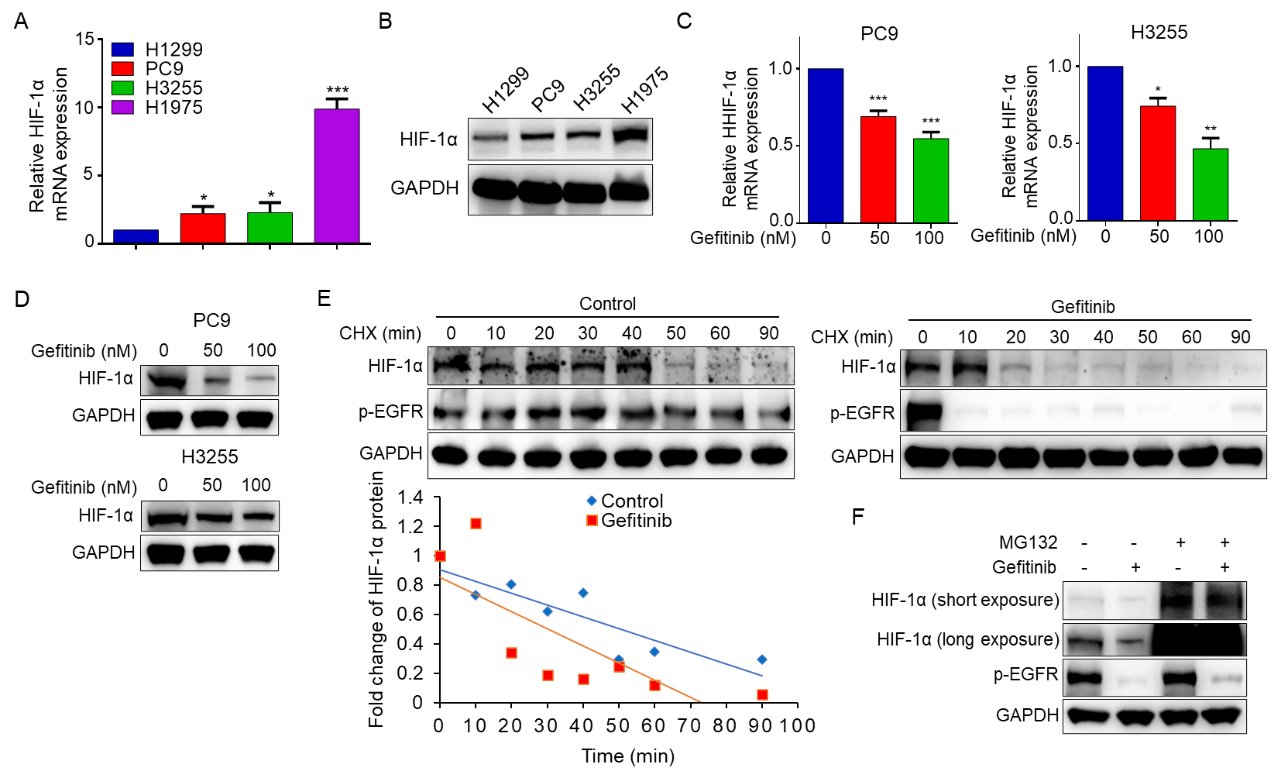


**Fig. S3** Gefitinib regulates HIF-1α expression by accelerating protein degradation. **A-B** The expression of HIF-1α in EGFR Mut LUAD cells and EGFR WT LUAD cells by real-time PCR and western blot. **C-D** RNA and protein expression of HIF-1α in PC9 and H3255 cells with gefitinib treatment by real-time PCR and western blot. **E** Protein expression of HIF-1α in PC9 cells without or with gefitinib (0.5 μM) were treated with 10 μM cycloheximide (CHX) for different time points. **F** HIF-1α expression in PC9 cells which first treated with gefitinib (0.5 μM) for 1 h, and then treated with MG132 (10 μM) for 3 h. Total proteins were lysed and detected by western blot. * *p* < 0.05, ** *p* < 0.01, *** *p* < 0.001.


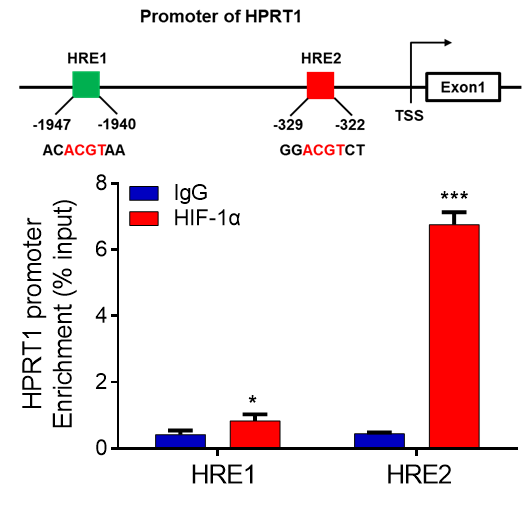


**Fig. S4** qChIP assay in PC9 cells. A higher proportion of the binding is shown in HIF-1α with HRE2 than with HRE1, IgG as the control. * *p* < 0.05, *** *p* < 0.001.


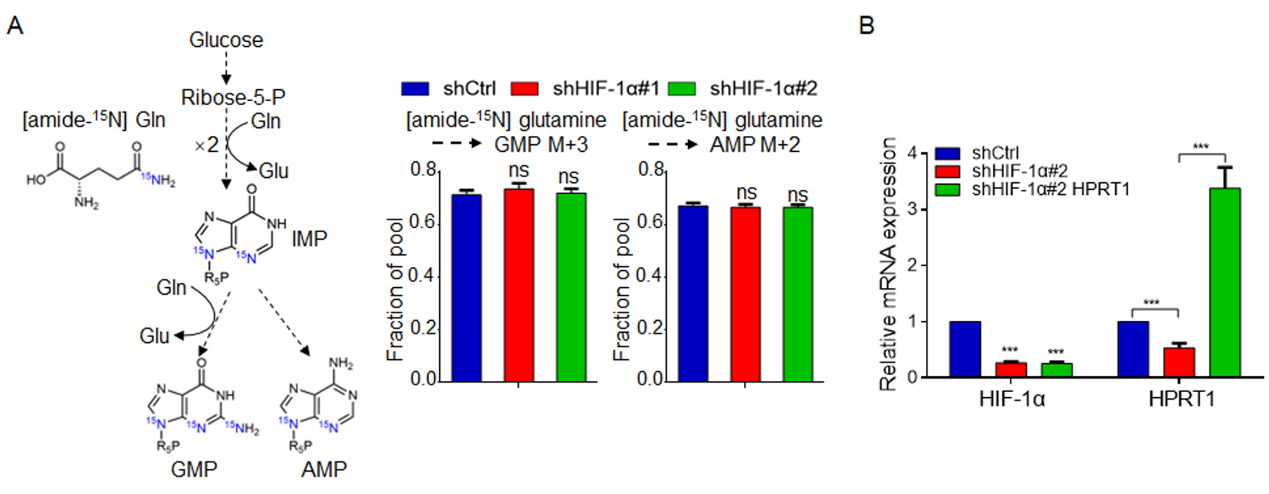


**Fig. S5** HIF-1α does not affect de novo purine synthesis. **A** Schematic illustrating amide-^15^N labeling of nucleotides from amide-^15^N-glutamine (left), amide-^15^N-glutamine-labeled purine metabolism intermediates examined by CE-TOF/MS in PC9 cells transfected with or without HIF-1α knockdown plasmids (right). ns, no significant. **B** The expression of HIF-1α and HPRT1 analyzed in PC9-shCtrl, PC9-shHIF-1α and PC9-shHIF-1α HPRT1 cells by real-time PCR. *** *p* < 0.001.
